# Factors influencing participation in lung cancer screening among screening-eligible adults: a scoping review

**DOI:** 10.1016/j.pmedr.2026.103585

**Published:** 2026-07-23

**Authors:** Cynthia Schneider, Luca Bosso, Louis Gros, Marie-Anne Durand, Kevin Selby, for the L-Factors Study Group, Kevin Ten Haaf, Dominik Menges, Chiara Pozzessere, Jean-Luc Bulliard, Christophe von Garnier, Milo Puhan, Christina Akre, Cédric Bongard, Andrea Bordoni, Romain Freund, Joachim Marti, Anna Nicolet, Mélinée Schindler

**Affiliations:** 1 Erasmus University Medical Center Rotterdam (Erasmus MC), Department of Public Health, Rotterdam, the Netherlands; 2 University of Zurich (UZH), Epidemiology, Biostatistics and Prevention Institute, Zürich, Switzerland; 3 Lausanne University Hospital (CHUV) and University of Lausanne, Department of Radiology, Lausanne, Switzerland; 4 University Center for Primary Care and Public Health, Unisanté, Department of Epidemiology and Health Systems, Lausanne, Switzerland; 5 Lausanne University Hospital (CHUV) and University of Lausanne, Division of Pulmonology, Department of Medicine, Lausanne, Switzerland; 6 Department of Health and Social Affairs, Cantonal Cancer Registry, Locarno, Switzerland; 7 University Center for Primary Care and Public Health, Unisanté, Cancer Screening Programs in Vaud, Lausanne, Switzerland; a Unisanté, University Centre for Primary Care and Public Health, Department of Ambulatory Care, Lausanne, Switzerland; b Lausanne University Hospital (CHUV) and University of Lausanne, Division of Oncology, Department of Medicine, Lausanne, Switzerland; c The Dartmouth Institute for Health Policy & Clinical Practice, Dartmouth College, Hanover, NH, United States of America.

**Keywords:** Lung cancer screening, Participation, Uptake, Barriers, Facilitators, Scoping review, COM-B

## Abstract

**Objective:**

Participation in low-dose computed tomography (LDCT) lung cancer screening remains suboptimal, and evidence on participation determinants is heterogeneous. This scoping review mapped participation factors using the Capability–Opportunity–Motivation–Behavior (COM—B) framework to guide intervention development.

**Methods:**

We conducted an international scoping review of studies published between January 2013 and June 2025. PubMed, Embase, CINAHL, PsycINFO, Web of Science, and the Cochrane Library were searched. Quantitative, qualitative, and mixed-methods studies examining participation among adults eligible for LDCT screening were included. Factors were coded inductively and mapped to framework domains. Overall, 3229 unique records were screened.

**Results:**

Thirty-nine studies were included: 22 quantitative, 13 qualitative, and four mixed-methods studies. Determinants spanned all framework domains. Factors most often associated with higher participation included risk awareness (*n* = 15), perceived benefits (*n* = 13), and clinician recommendation (*n* = 12). Factors most often associated with lower participation included misconceptions (n = 13), competing priorities (n = 12) and fear of results (*n* = 11).

**Conclusions:**

Participation reflects interacting factors. Health literacy barriers, misconceptions, and practical constraints may prevent favorable intentions from translating into uptake. Interventions should combine tailored communication, clinician engagement, improved access, and support addressing risk perception, fear, stigma, and trust.

## Highlights

• Participation depends on individual, healthcare and emotional factors
• Knowledge gaps and misconceptions are frequent participation barriers
• Clinician recommendation and supportive pathways facilitate uptake
• Fear, stigma and trust shape decisions beyond eligibility awareness
• Multicomponent strategies may reduce the intention–behavior gap

## 1 Introduction

Lung cancer remains a major public health burden in Europe, accounting for approximately one in five cancer deaths, 250,000 deaths and more than three million disability-adjusted life years lost each year (ECIS - European Cancer Information System, 2025). Low-dose computed tomography (LDCT) screening is recommended for individuals at high risk based on age and smoking history (US Preventive Services Task Force, 2021), and reduces lung cancer mortality, particularly among long-term current and former smokers aged 50–80 years (de Koning et al., 2020). Multiple countries have launched pilot screening programs or are implementing organized lung cancer screening.

Despite demonstrated clinical effectiveness, participation in LDCT screening remains suboptimal, even in programs that facilitate access or provide screening at no direct cost (Crosbie et al., 2019; Jungblut et al., 2023). Low and socially patterned uptake, particularly among socioeconomically disadvantaged populations, threatens population-level impact of screening and risks widening health inequalities. Understanding why eligible individuals participate, or decline to participate, is therefore essential to improving equity and optimizing program design (Byrne et al., 2019).

Participation in preventive health programs is shaped by individual, social, and structural influences, including health literacy, perceived risk, beliefs, emotions, access, service organization, and delivery pathways (Broekhuizen et al., 2017; Norman et al., 2020; Schindler et al., 2026). These multilevel dynamics are particularly salient in lung cancer screening, where screening-eligible populations are disproportionately affected by social disadvantages and structural barriers to care (Gros et al., 2026). Preference-elicitation studies further suggest that individuals make trade-offs between perceived benefits, potential harms, and program features, indicating that participation is influenced by multiple interacting factors rather than a single barrier (Byrne et al., 2019; Broekhuizen et al., 2017; Norman et al., 2020). However, the evidence remains challenging to synthesize because studies vary in outcomes, contexts, and designs. Existing reviews have summarized barriers and facilitators to lung cancer screening participation (Belachew et al., 2025; Salman et al., 2025), but less attention has been given to integrating qualitative, quantitative, and preference-based evidence within a behavioral framework designed to inform intervention development.

The Capability–Opportunity–Motivation–Behavior framework provides such an approach by organizing determinants of behavior into three interacting domains: capability, opportunity, and motivation (Michie et al., 2011). Capability refers to physical and psychological capacity, including knowledge and understanding. Opportunity refers to external physical and social conditions, such as access, affordability, service organization, clinician communication, and social support. Motivation refers to reflective and automatic processes, including beliefs, perceived risk, emotions, stigma, trust, intentions, and habits. These domains can be further divided into six subdomains: physical and psychological capability, physical and social opportunity, and reflective and automatic motivation.

Scoping review methodology is especially appropriate when literature is heterogeneous in terms of design, populations, and reported constructs, and when the aim is to map the breadth of available evidence rather than estimate effect sizes (Arksey and O'Malley, 2005; Peters et al., 2020; Levac et al., 2010). Accordingly, this scoping review aimed to identify and map factors influencing participation in, or intention to participate in (hereafter referred to as participation factors), LDCT lung cancer screening among eligible adults across organized, pilot, and opportunistic screening contexts. Findings are organized using the COM-B model to provide a theory-informed synthesis to inform equitable intervention design.

## 2 Methods

### 2.1 Study design and reporting

This scoping review was conducted using an established scoping review approach aligned with the Joanna Briggs Institute scoping review guideline (Peters et al., 2020) and reported in accordance with the Preferred Reporting Items for Systematic Reviews and Meta-Analyses (PRISMA) - Extension for Scoping Reviews (ScR) (Tricco et al., 2018) (Supplementary File S1. PRISMA-ScR checklist). The review protocol was prospectively registered on the Open Science Framework on 8 July 2025 (Schneider et al., 2025).

### 2.2 Eligibility criteria

Eligibility criteria were defined a priori using the Population–Concept–Context framework, a structured approach recommended for scoping reviews to guide inclusion criteria (Peters et al., 2020).

*Population***:** We included studies involving adults eligible for LDCT lung cancer screening, typically defined as individuals aged 50–80 years with a significant smoking history, in accordance with major screening trials and guidelines. Studies using alternative but comparable eligibility criteria were also included if participants were clearly identified as screening-eligible.

*Concept***:** We included empirical evidence on factors influencing screening participation, including barriers, facilitators, determinants, beliefs, attitudes, perceptions, and structural or logistical influences related to initial uptake (e.g., intention, attendance or enrolment).

*Context***:** We included evidence from organized screening programs, pilot programs, trials, and opportunistic screening contexts, provided participants were screening-eligible and the study examined participation-related factors. Studies from all geographical regions and healthcare systems were considered.

*Other criteria.* We included empirical quantitative, qualitative, and mixed-methods studies published between 2013 and 2025, in English or French. Studies were included regardless of country. Grey literature, including conference abstracts, editorials, commentaries, and protocols, was excluded. Studies focusing exclusively on healthcare professionals or policymakers (without reporting factors from the perspective of screening-eligible individuals or measured determinants of uptake among eligible individuals) were excluded.

### 2.3 Information sources and search strategy

A comprehensive search strategy was developed and iteratively refined with support from the study team and an information specialist. Electronic searches from January 2013 to June 2025 were conducted in six databases: PubMed (MEDLINE), Embase, CINAHL, PsycINFO, Web of Science, and the Cochrane Library. The search was limited to studies published from January 2013 onward to capture evidence generated following the publication of major randomized trials demonstrating the effectiveness of LDCT screening and the subsequent implementation of screening programs in several countries. Reference list screening and citation tracking were conducted for included papers and relevant reviews; these searches did not identify additional eligible records beyond those retrieved through database searches.

Search strategies combined database-specific controlled vocabulary and keywords related to lung cancer, LDCT screening, and participation-related constructs. Terms included combinations of “lung cancer,” “lung neoplasms,” “low-dose computed tomography,” “LDCT screening,” “lung cancer screening,” “screening program,” “participation,” “uptake,” “attendance,” “adherence,” “intention,” “barriers,” “facilitators,” “determinants,” “preferences,” “decision making,” “motivation,” “invitation,” “access,” “healthcare access,” “service delivery,” and “structural barriers.” Searches were limited to English- or French-language publications. The full electronic search strategy is provided in Supplementary File S2.

### 2.4 Selection of sources of evidence

All records were exported to Zotero® for reference management and deduplication. Deduplicated records were then imported into Rayyan® for screening.

**Fig. 1:**
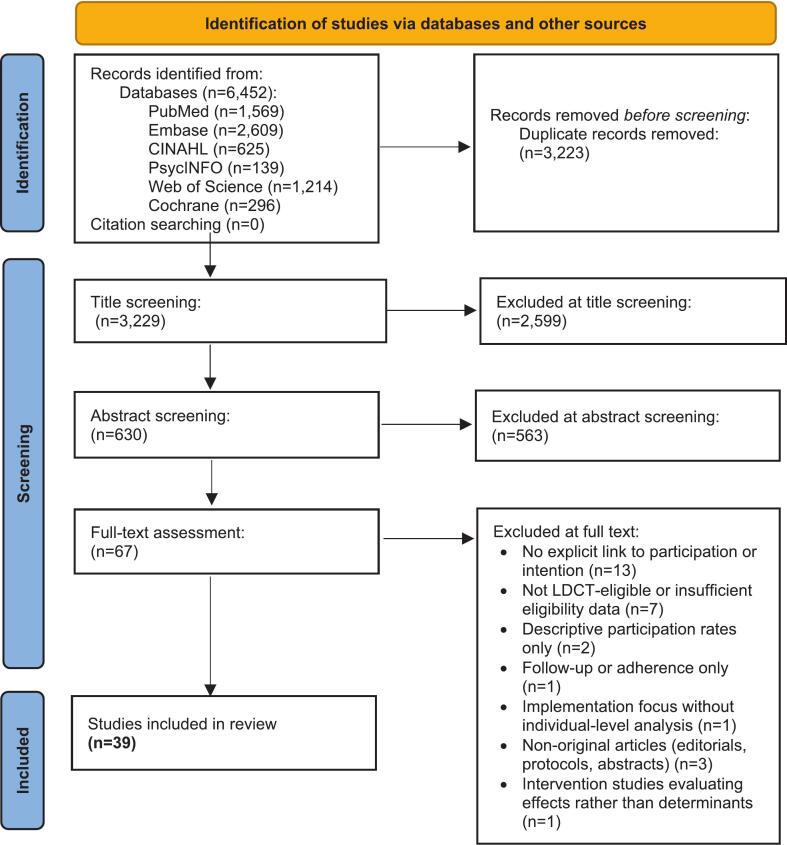
Preferred Reporting Items for Systematic Reviews and Meta-Analyses extension for Scoping Reviews (PRISMA) Flow diagram of study identification, screening, eligibility assessment, and inclusion for the scoping review of factors influencing participation in lung cancer screening among screening-eligible adults, January 2013–June 2025.

Study selection followed a multi-stage screening process: [1] title screening, [2] title/abstract screening, and [3] full-text screening. Screening was conducted independently by three authors, with disagreements resolved through discussion and consultation with a fourth reviewer when needed. Reasons for full-text exclusion were documented, and the study selection process is summarized in a PRISMA-ScR flow diagram (Fig. 1). Consistent with the scoping review methodology, articles were not subjected to critical appraisal of quality.

### 2.5 Data extraction

Data were charted using a structured extraction form that was piloted and refined before full extraction. For each included study, we extracted:

• bibliographic information (author, year) and country/setting
• study design and aims
• sample characteristics and screening eligibility definition
• screening context (e.g., program, pilot, trial, opportunistic)
• outcome focus (e.g., intention, uptake/attendance, adherence)
• all reported participation-related factors, including how they were described by participants and/or operationalized by study authors (e.g., themes, reasons, measured predictors)
• stage of the participation pathway (e.g., intention/decision-making; scheduling/attendance; follow-up/adherence), when the primary studies provided sufficient information.

Participation-related factors were extracted as reported by the original study authors. In quantitative studies, these corresponded to variables examined as predictors or correlates of participation outcomes (e.g., statistically associated with screening uptake). In qualitative studies, factors corresponded to themes or explanations identified by participants or interpreted by study authors as influencing participation decisions.

Data extraction was conducted by one reviewer and checked by a second reviewer for completeness and consistency.

### 2.6 Data analysis and synthesis

Data analysis followed a structured and iterative coding process, consistent with methodological guidance for scoping reviews. Extracted factors were first coded inductively in MaxQDA®, without applying a predefined theoretical structure. This step aimed to capture the range and diversity of reported factors influencing participation in LDCT lung cancer screening.

The coded factors were then mapped onto the COM-B framework (Michie et al., 2011). Participation in lung cancer screening was defined as the target behavior. The framework was used as an organizing structure to classify and present findings, rather than as an explanatory model or an inclusion criterion imposed on primary studies.

Each factor was assigned to the Capability, Opportunity, or Motivation domain and, where applicable, to one of the six framework subdomains: physical capability, psychological capability, physical opportunity, social opportunity, reflective motivation, or automatic motivation. Classification was based on the primary behavioral mechanism described in the original study. Factors describing sociodemographic characteristics, such as age, sex, or race/ethnicity, were summarized separately as non-COM-B factors.

Each factor was also coded according to its reported direction of association with participation. Direction reflected whether the factor was associated with higher participation, lower participation, or showed mixed or context-dependent associations across studies. Coding decisions were discussed among reviewers to ensure conceptual consistency and transparent classification.

## 3 Results

### 3.1 Summary of included studies

A total of 6452 records were identified through database searching, of which 3229 remained after deduplication and were screened for eligibility. A total of 39 studies were included in this scoping review (Fig. 1). Most studies were conducted in North America (*n* = 26), and nearly half published between 2023 and 2025 (*n* = 17). Designs were heterogeneous (quantitative *n* = 22; qualitative *n* = 13; mixed methods *n* = 4). Outcomes most frequently focused on actual uptake (n = 17) and intention to participate (*n* = 14). Aggregated study characteristics are summarized in Table 1, and study-level characteristics and main findings are provided in Table 2. Complete references for the included studies are provided in Supplementary File S3.

**Table 1:** Aggregated characteristics of the 39 studies included in the scoping review of factors influencing participation in lung cancer screening among screening-eligible adults, published between January 2013 and June 2025.

Dimension	Subcategory	Number of studies (N)
Region	North America	26
	Asia	7
	Europe	4
	Oceania	2
Year of publication	2015–2017	3
	2018–2020	8
	2021–2022	11
	2023–2025	17
Study design	Quantitative	22
	Qualitative	13
	Mixed methods	4
Screening context	Organized	14
	Opportunistic	14
	Hypothetical	7
	Pilot/ trial	4
Sample size range	< 50 participants	13
	50–500 participants	7
	500–5000 participants	11
	> 5000 participants	8
Participation focus	Intention (hypothetical participation)	14
	Actual uptake	17
	Follow-through after referral	8

*Notes: N* *=* *number.*

**Table 2:** Study-level characteristics and summarized findings of the 39 studies included in the scoping review of factors influencing participation in lung cancer screening among screening-eligible adults, published between January 2013 and June 2025.

Study ID	Citation	Country	Screening context	Population characteristics	Sample size (n)	Design	Main findings (mapped factors)
S1	Alaniz-Cantú et al., 2024	USA	Opportunistic	Latino adults mean age 58; eligible 50–80 years; ≥20 pack-year; current/quit ≤15 years	20	QUAL	Awareness of lung cancer screening and eligibility was low. Participation was facilitated by perceived benefits of early detection, peace of mind, self-care, and smoking cessation. Barriers included fear of results, cost, lack of clinician recommendation, limited knowledge of the procedure, time constraints, transportation difficulties, and self-blame. Family encouragement, clinician recommendation, symptoms, and accessible educational materials acted as cues to action. Cultural and social context strongly shaped decisions.
S2	Ali et al., 2015	UK	Pilot/ trial	High-risk adults aged 50–75 years identified via risk algorithm; current and former smokers	8729	MIXED	Non-uptake was more frequent among women, older adults, current smokers, and individuals with lower socioeconomic status. Practical barriers (transport, cost, comorbidities, competing commitments) were most common, followed by emotional barriers such as fear, anxiety, and avoidance. Low perceived risk and dislike of hospitals or scans also discouraged participation.
S3	Anderson et al., 2023	USA	Opportunistic	Indigenous adults aged 45–79 years; current or former smokers	24	QUAL	Participation was influenced by knowledge of screening, access to resources, trust in healthcare, fear or ambivalence about results, and cultural beliefs. Trust in community clinics and family motivation facilitated screening, while misconceptions and limited clinician time acted as barriers. Culturally tailored education, community-based outreach, and integrating screening into routine care were identified as key facilitators.
S4	Cao et al., 2022	China	Organized	High-risk adults aged 40–74 years identified via risk assessment; current, former and non-smokers included	221,955	QUANT (prospective cohort)	Uptake was higher among women, former smokers, individuals with comorbidities or family history of lung cancer, and those exposed to media campaigns or navigation support. Barriers included current smoking, depressive symptoms, lack of awareness, preference not to know results, and long delays between referral and screening. Structural factors such as appointment delays and lack of navigation substantially reduced uptake.
S5	Carter-Bawa et al., 2024	USA	Organized	Screening-eligible adults aged 50–80 years, ≥20 pack-year; current/quit ≤15 years; current and former smokers	529	QUANT (observational cohort)	Higher knowledge, self-efficacy, and perception of shared decision-making were associated with screening participation and completion. Older age and stronger self-efficacy predicted opting into screening. Perceived barriers and benefits influenced decisional quality in complex ways, highlighting the role of psychosocial factors beyond simple access.
S6	Carter-Harris et al., 2017	USA	Opportunistic	Screening-eligible adults aged 55–77 years; current and former smokers (≥30 pack-year)	18	QUAL	Preferences for screening were similar across racial groups, but completion was lower among African American participants. Worry, perceived susceptibility, and social influence shaped decisions, while structural and systemic barriers explained disparities in completion despite comparable preferences.
S7	Carter-Harris et al., 2018	USA	Opportunistic	Screening-eligible long-term smokers aged 55–80 years; current and former smokers (≥30 pack-year)	438	QUANT (cross-sectional)	Physician recommendation was central to screening participation; no participants screened against clinician advice. Barriers included lack of symptoms, misconceptions about screening necessity, cost concerns, fear of results, and competing priorities. Pulmonary symptoms, valuing early detection, and insurance coverage motivated participation.
S8	Cho & Cho, 2024	South Korea	Opportunistic	High-risk adults aged 50–74 years; ≥20 pack-year (current/quit ≤15 years; current and former smokers	186	QUANT (cross-sectional)	Motivation to screen was generally high, but self-efficacy distinguished screeners from decliners. Barriers included limited understanding of screening purpose, logistical challenges related to travel and cost, lack of family support, and competing responsibilities. Family encouragement, belief in early detection, and perceived personal control facilitated participation.
S9	Colhoun et al., 2024	New Zealand	Hypothetical	Māori current or former smokers aged 50–75 years and whānau members	30	QUAL	Screening uptake was strongly associated with insurance coverage, access to primary care, and presence of COPD. Individuals without insurance or a regular provider were substantially less likely to participate. Marked geographic variation suggested system-level differences in access and implementation.
S10	Copeland et al., 2023	USA	Opportunistic	Mostly Black adults from rural and underserved Alabama; screening-eligible current or former smokers aged 55–80 years (≥30 pack-year)	58	QUAL	Participation was higher among women, individuals with higher education, former smokers, and those with a family history of cancer. Current smoking and lower education were associated with lower uptake. Preventive health behaviors and family health history played an important role in participation decisions.
S11	Couraud et al., 2018	France	Pilot/ trial	Current and former smokers aged 40–75 years; former smokers quit <15 years; majority <30 pack-year	1463	QUANT (cross-sectional)	Although most participants perceived lung cancer screening as beneficial, actual participation was low. Barriers included cost, lack of symptoms, fear of positive results, and downstream financial concerns. Preferences favored reminders, clinician involvement, and clear educational materials, particularly among individuals with lower self-efficacy or financial hardship.
S12	Daskalakis et al., 2023	USA	Organized	Screening-eligible adults aged 50–80 years; current and former smokers (≥20 pack-year); racially diverse (White/Black)	419	QUANT (observational)	Most respondents supported offering lung cancer screening and expressed willingness to participate, particularly those meeting eligibility criteria. Reasons for refusal included fear of knowing results, feeling too old, lack of symptoms, and disbelief in early detection benefits. Many eligible smokers expressed interest in receiving smoking cessation support alongside screening.
S13	Draucker et al., 2019	USA	Opportunistic	Screening-eligible long-term smokers aged 55–70 years; current and former smokers (≥30 pack-year)	39	QUAL	Higher intention to screen was associated with future-oriented time preferences and absence of present bias. Lower income and present-focused decision-making were linked to reduced intention. These findings highlight the role of temporal preferences and socioeconomic factors in screening decisions.
S14	Dunlop et al., 2022	Australia	Pilot/ trial	High-risk adults aged 55–80 years; current and former smokers (≥30 pack-year or high PLCOm2012 risk)	39	QUAL	Uptake was higher among individuals insured through Medicaid and those living within the screening service area, while Medicare coverage and out-of-pocket payment were associated with lower uptake. Older age facilitated participation, whereas access-related factors largely explained observed disparities.
S15	Gudina et al., 2023	USA	Opportunistic	Screening-eligible adults aged 55–80 years per USPSTF 2013; current and former smokers (≥30 pack-year)	11,297	QUANT (cross-sectional)	Participation was higher among individuals living in urban areas, those previously screened, and those exposed to media information. Higher knowledge, perceived risk, and positive beliefs about screening facilitated uptake, while social influence from peers sometimes discouraged participation.
S16	Guo et al., 2022	China	Organized	Urban adults aged 40–74 years; high-risk for lung cancer per CanSPUC risk score; current, former and never smokers included	55,428	QUANT (cross-sectional)	Knowledge of lung cancer screening was low even among screened individuals. Trust in providers and social support facilitated participation, while fear of false positives, ambivalence, and difficulty navigating centralized processes acted as barriers. Repeated communication and simplified pathways were suggested to improve uptake.
S17	Jung et al., 2024	USA	Opportunistic	Screening-eligible adults aged 50–80 years; current and former smokers (≥20 pack-year)	86	QUANT (cross-sectional)	Screening completion was lower among Black veterans, older individuals, current smokers, and those with mental health or substance use disorders. Connection with a program nurse was a critical step, yet many individuals never established this contact, contributing to non-completion.
S18	Kellen et al., 2021	Belgium	Hypothetical	Current and former smokers aged ≥18 years from the general population; subgroup fitting NELSON criteria	1534	QUANT (cross-sectional)	Completion of ordered screening was associated with older age, former smoking, COPD, centralized program organization, and recent smoking cessation treatment. Lower neighborhood socioeconomic status, unknown insurance, and current smoking reduced completion, highlighting the importance of system design and continuity.
S19	Lee et al., 2020	South Korea	Hypothetical	General population 20–74 years; analyses of NLST/NELSON-eligible ever-smokers (national survey).	4500	QUANT (cross-sectional)	Overall screening uptake was extremely low. Participation was facilitated by comorbidities, current smoking, closer distance to screening centers, and dual insurance coverage. Barriers included older age, greater distance, lower income, and reliance on public insurance alone, with differences across healthcare systems.
S20	Li et al., 2021	USA	Organized	Screening-eligible adults aged 55–80 years; current and former smokers (≥30 pack-year)	1355	QUANT (cross-sectional)	Positive beliefs about early detection, personal control, treatment effectiveness, and smoking cessation increased screening uptake. Negative emotional representations such as fear and fatalism also influenced decisions in complex ways. Psychological perceptions were central determinants of participation.
S21	Lin et al., 2024	China	Opportunistic	High-risk adults aged 50–74 years per Chinese guidelines; current, former and non-smokers included	1652	QUANT (cross-sectional)	Non-participation was associated with female sex, current smoking, lower cumulative smoking exposure, and certain comorbidities. Commonly reported barriers included unawareness of appointments, distance, insurance concerns, and fear of results, alongside substantial knowledge gaps regarding benefits and harms.
S22	Navuluri et al., 2023	USA	Organized	Black Veterans aged ∼68 years; current and former smokers (≥30 pack-year)	32	QUAL	Lack of knowledge about lung cancer screening was the most consistent barrier, even after referral. Competing health priorities, scheduling complexity, fear of bad news, and discomfort with imaging discouraged participation. Trust in primary care clinicians and understanding the benefits of early detection facilitated uptake.
S23	Navuluri et al., 2023	USA	Organized	Veterans aged ∼50–80 years; current and former smokers (≥20 pack-year); Black and White veterans	4562	QUANT (retrospective cross-sectional)	Screening decisions were shaped by intersecting environmental, psychosocial, and structural factors, including racism, mistrust, fear, financial instability, and transportation barriers. Clear, empathetic communication and reassurance about the non-invasive nature and coverage of screening supported participation.
S24	Neslund-Dudas et al., 2023	USA	Organized	Screening-eligible adults aged 55–80 years; current and former smokers (≥20 pack-year); multisite	18,294	QUANT (retrospective cohort)	Willingness to participate was driven by disease cognition, perceived risk, family cancer history, and smoking status. Individuals with low knowledge or low affective risk perception were less willing to screen, particularly within moderate-risk groups where disease understanding was pivotal.
S25	Niranjan et al., 2025	USA	Organized	Screen-eligible adults aged 50–80 years; current and former smokers (≥20 pack-year); predominantly underserved populations; urban and rural residents	67,355	QUANT (retrospective cohort)	Participation was motivated by trust in clinicians, belief in early detection benefits, low perceived harms, and personal experiences with cancer among family or friends. Most participants were unaware of lung cancer screening prior to clinician referral.
S26	Quaife et al., 2021	UK	Pilot/ trial	Ever-smokers aged 55–77 years current and former smokers; socioeconomically diverse	7730	QUANT (prospective longitudinal cohort)	Screening decisions were strongly influenced by material conditions such as housing stability, income, and access to non-judgmental care. Supportive provider relationships facilitated participation, while stigma, competing survival priorities, and negative institutional experiences discouraged engagement.
S27	Raju et al., 2020	USA	Organized	Screening-eligible adults aged ∼55–80 years; current and former smokers; mean 50 pack-year.	818	MIXED (case–control + survey)	Barriers included lack of knowledge, absence of provider recommendation, transportation difficulties, and fear of results. Provider endorsement and community-based outreach were viewed as powerful facilitators, particularly in rural settings.
S28	Richman et al., 2022	USA	Organized	Screening-eligible adults aged ∼55–80 years; current and former smokers; racially diverse	16	QUAL	Screening participation was higher among individuals with COPD and among women reporting poorer health status. Men without COPD and individuals without a regular primary care provider had particularly low uptake, underscoring the role of clinical engagement.
S29	Richmond et al., 2024	USA	Hypothetical	Screening-eligible adults aged 50–80 years; current and former smokers (mean ∼ 39 pack-year); racially diverse sample	34	QUAL	Among Korean immigrant men, screening was facilitated by provider and family recommendations, prior experiences with preventive care, and existing health concerns. Barriers included cost, lack of time, limited knowledge, misconceptions about lung cancer, and absence of clinician recommendation within the U.S. healthcare system.
S30	Rong et al., 2023	China	Opportunistic	High-risk adults aged 50–75 years; current and former smokers (≥30 pack-year) and long-term passive smokers.	1955	QUANT (cross-sectional)	Gaining Medicare coverage at age 65 increased screening uptake among high-risk men and reduced cost-related care avoidance. However, most eligible individuals remained unscreened, indicating persistent non-financial barriers.
S31	Roth et al., 2018	USA	Organized	Screening-eligible adults aged 55–77 years; current and former smokers (≥30 pack-year).	20	QUAL	Completion of lung cancer screening was associated with eligibility, social support, former smoking, emphysema, and family history. Knowledge gaps, low perceived risk, misperceptions, and attending decision-making visits alone were key contributors to non-completion.
S32	Sayani et al., 2021	Canada	Organized	High-risk adults aged 55–74 years living with low income; long-term smokers (≥20 years).	18	QUAL	Screening utilization was higher among insured individuals and those with respiratory comorbidities. Substantial variation across states suggested differences in access and healthcare delivery rather than individual characteristics alone.
S33	Schiffelbein et al., 2020	USA	Hypothetical	Rural screening-eligible adults aged 55–75 years; current and former smokers (≥20 pack-year).	23	MIXED (concurrent embedded; primarily QUAL)	Preferences favored annual screening, hospital-based services, and combined testing, while higher out-of-pocket costs and lack of insurance increased opt-out rates. Risk perception and preventive health habits further shaped screening choices.
S34	Sedani et al., 2021	USA	Opportunistic	Screening-eligible adults aged 55–80 years; current and former smokers (≥20 pack-year) high-burden tobacco state (Oklahoma).	596	QUANT (cross-sectional)	Awareness of lung cancer screening and eligibility was low. Participation was facilitated by perceived benefits of early detection, peace of mind, self-care, and smoking cessation. Barriers included fear of results, cost, lack of clinician recommendation, limited knowledge of the procedure, time constraints, transportation difficulties, and self-blame. Family encouragement, clinician recommendation, symptoms, and accessible educational materials acted as cues to action. Cultural and social context strongly shaped decisions.
S35	Sin et al., 2016	USA	Hypothetical	Korean immigrant men aged 55–79 years; current and former smokers (≥30 pack-year); Korean-speaking.	24	QUAL	Non-uptake was more frequent among women, older adults, current smokers, and individuals with lower socioeconomic status. Practical barriers (transport, cost, comorbidities, competing commitments) were most common, followed by emotional barriers such as fear, anxiety, and avoidance. Low perceived risk and dislike of hospitals or scans also discouraged participation.
S36	Sun et al., 2022	USA	Opportunistic	High-risk adults aged ∼56–74 years aged 50–80 years; current and former smokers (≥20 pack-year); nationally representative sample.	11,163	QUANT (quasi-experimental)	Participation was influenced by knowledge of screening, access to resources, trust in healthcare, fear or ambivalence about results, and cultural beliefs. Trust in community clinics and family motivation facilitated screening, while misconceptions and limited clinician time acted as barriers. Culturally tailored education, community-based outreach, and integrating screening into routine care were identified as key facilitators.
S37	Wong et al., 2024	USA	Organized	Screening-eligible adults aged 50–80 years; current and former smokers (median ∼ 30 pack-year).	380	MIXED (cross-sectional)	Uptake was higher among women, former smokers, individuals with comorbidities or family history of lung cancer, and those exposed to media campaigns or navigation support. Barriers included current smoking, depressive symptoms, lack of awareness, preference not to know results, and long delays between referral and screening. Structural factors such as appointment delays and lack of navigation substantially reduced uptake.
S38	Zahnd & Eberth, 2019	USA	Opportunistic	Screening-eligible adults aged 55–80 years; current and former smokers (≥30 pack-year).	4373	QUANT (cross-sectional)	Higher knowledge, self-efficacy, and perception of shared decision-making were associated with screening participation and completion. Older age and stronger self-efficacy predicted opting into screening. Perceived barriers and benefits influenced decisional quality in complex ways, highlighting the role of psychosocial factors beyond simple access.
S39	Zhao et al., 2021	China	Hypothetical	High-risk adults aged 50–74 years; ever-smokers (current/former) and individuals with passive smoking, occupational exposure, COPD/emphysema, or family history.	392	QUANT (Discrete Choice Experiment)	Preferences for screening were similar across racial groups, but completion was lower among African American participants. Worry, perceived susceptibility, and social influence shaped decisions, while structural and systemic barriers explained disparities in completion despite comparable preferences.

Notes: Study IDs refer to the supplementary reference list of included studies. USA = United States of America; UK = United Kingdom; LDCT = low-dose computed tomography; USPSTF = U.S. Preventive Services Task Force; QUAL = qualitative; QUANT = quantitative; MIXED = mixed methods; PLCOm2012 = Prostate, Lung, Colorectal and Ovarian Cancer Screening Trial 2012 risk prediction model; CanSPUC = Cancer Screening Program in Urban China risk assessment model; NLST = National Lung Screening Trial; NELSON = Nederlands–Leuvens Longkanker Screenings Onderzoek; COPD = Chronic Obstructive Pulmonary Disease.

**Fig. 2:**
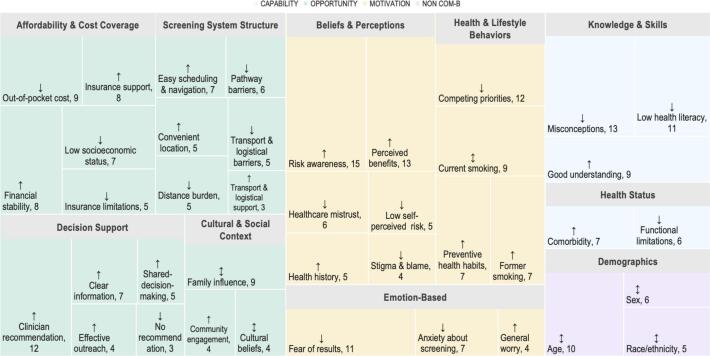
Treemap showing the distribution and reported direction of association of factors influencing lung cancer screening participation across Capability–Opportunity–Motivation–Behavior (COM-B) framework domains among the 39 studies included in the scoping review, published between January 2013 and June 2025. Notes:; ↑ = associated with higher participation; ↓ = associated with lower participation; ↕ = mixed or context-dependent association; Number = studies reporting each factors.

The following sections describe factors reported across the included studies within each COM-B domain and their corresponding subdomains. The detailed mapping of factors to individual studies is presented in Table 3. Fig. 2 summarizes the distribution of reported participation factors across COM-B domains, together with their reported direction of association with participation.

**Table 3:** Evidence map showing the distribution of reported factors influencing lung cancer screening participation among screening-eligible adults, organized according to Capability, Opportunity, and Motivation domains of the Capability–Opportunity–Motivation–Behavior (COM–B) framework, across the included studies (2013–2025).

COM-B domain	Com-B subdomain	Theme & Subtheme	S1. Alaniz-Cantú et al., 2024	S2. Ali et al., 2015	S3. Anderson et al., 2023	S4. Cao et al., 2022	S5. Carter-Bawa et al., 2024	S6. Carter-Harris et al., 2017	S7. Carter-Harris et al., 2018	S8. Cho & Cho, 2024	S9. Colhoun et al., 2024	S10. Copeland et al., 2023	S11. Couraud et al., 2018	S12. Daskalakis et al., 2023	S13. Draucker et al., 2019
CAPABILITY (n=31)	Physical Capability	1. Health Status													
		1.1 Comorbidity				✓									
		1.2 Functional limitations		✓		✓									
	Psychological Capability	2. Knowledge & Skills													
		2.2 Good understanding	✓				✓				✓		✓		✓
		2.3 Low health literacy	✓	✓	✓			✓							✓
		2.4 Misconceptions		✓	✓			✓			✓				✓
OPPORTUNITY (n=34)	Physical Opportunity	3. Affordability & Cost Coverage													
		3.1 Insurance limitations						✓	✓						
		3.2 Insurance support													✓
		3.3 Low socioeconomic status		✓	✓				✓						
		3.4 Out-of-pocket cost	✓								✓	✓			✓
		3.5 Financial stability				✓							✓		
		4. Screening system structure													
		4.1 Pathway barriers			✓						✓				
		4.2 Convenient location			✓										
		4.3 Distance burden		✓							✓				
		4.4 Easy scheduling & navigation			✓	✓					✓				
		4.5 Transport & logistical barriers	✓		✓										
		4.6 Transport & logistical support			✓						✓				
		5. Decision Support													
		5.1 Effective outreach			✓										
		5.2 Clear information	✓		✓	✓					✓	✓			
	Social Opportunity	5.3 No clinician recommendation	✓												✓
		5.4 Shared decision-making			✓			✓							
		5.5 Clinician recommendation	✓		✓						✓	✓			
		6. Cultural & Social Context													
		6.1 Community engagement			✓							✓			
		6.2 Cultural beliefs			✓						✓			✓	
		6.3 Family influence	✓	✓								✓			
MOTIVATION (n=35)	Reflective Motivation	7. Beliefs & Perceptions													
		7.1 Risk awareness				✓				✓		✓		✓	✓
		7.2 Perceived benefits	✓		✓						✓		✓	✓	✓
		7.3 Health history				✓			✓						
		7.4 Stigma & blame	✓								✓				
		7.5 Healthcare mistrust		✓	✓						✓				
		7.6 Low self-perceived risk		✓									✓		
		8. Health & Lifestyle Behaviors													
		8.1 Preventive health habits					✓			✓					
		8.2 Competing priorities	✓	✓				✓			✓				✓
	Automatic Motivation	8.3 Current smoking		✓		✓							✓		
		8.4 Former smoking				✓							✓		
		9. Emotion-Based													
		9.1 Anxiety about screening		✓	✓			✓							
		9.2 Fear of results	✓	✓	✓			✓							✓
		9.3 General worry	✓							✓				✓	
NON COM-B (n=16)		10. Demographics													
		10.1 Sex		✓		✓							✓		
		10.2 Race/ethnicity							✓					✓	
		10.3 Age		✓			✓								
		THEME & subtheme	S14. Dunlop et al., 2022	S15. Gudina et al., 2023	S16. Guo et al., 2022	S17. Jung et al., 2024	S18. Kellen et al., 2021	S19. Lee et al., 2020	S20. Li et al., 2021	S21. Lin et al., 2024	S22. Navuluri et al., 2024	S23. Navuluri et al., 2023	S24. Neslund-Dudas et al., 2023	S25. Niranjan et al., 2025	S26. Quaife et al., 2021
CAPABILITY (n=31)	Physical Capability	1. Health Status													
		1.1 Comorbidity											✓	✓	
		1.2 Functional limitations									✓	✓			
	Psychological Capability	2. Knowledge & Skills													
		2.2 Good understanding								✓					✓
		2.3 Low health literacy									✓				
		2.4 Misconceptions	✓			✓	✓								
OPPORTUNITY (n=34)	Physical Opportunity	3. Affordability & Cost Coverage													
		3.1 Insurance limitations		✓					✓						
		3.2 Insurance support							✓			✓		✓	
		3.3 Low socioeconomic status					✓					✓	✓	✓	
		3.4 Out-of-pocket cost				✓									
		3.5 Financial stability			✓					✓		✓			
		4. Screening system structure													
		4.1 Pathway barriers		✓											
		4.2 Convenient location	✓						✓				✓		
		4.3 Distance burden	✓											✓	
		4.4 Easy scheduling & navigation	✓			✓		✓			✓				
		4.5 Transport & logistical barriers	✓												
		4.6 Transport & logistical support				✓									
		5. Decision Support													
		5.1 Effective outreach				✓				✓	✓				
		5.2 Clear information				✓					✓				
	Social Opportunity	5.3 No clinician recommendation													
		5.4 Shared decision-making									✓				
		5.5 Clinician recommendation									✓				
		6. Cultural & Social Context													
		6.1 Community engagement									✓				
		6.2 Cultural beliefs													
		6.3 Family influence	✓							✓	✓				
MOTIVATION (n=35)	Reflective Motivation	7. Beliefs & Perceptions													
		7.1 Risk awareness	✓				✓			✓	✓				✓
		7.2 Perceived benefits	✓												✓
		7.3 Health history			✓										
		7.4 Stigma & blame	✓												✓
		7.5 Healthcare mistrust													
		7.6 Low self-perceived risk		✓		✓									
		8. Health & Lifestyle Behaviors													
		8.1 Preventive health habits			✓										✓
		8.2 Competing priorities	✓					✓							
	Automatic Motivation	8.3 Current smoking			✓	✓						✓			
		8.4 Former smoking			✓							✓	✓	✓	
		9. Emotion-Based													
		9.1 Anxiety about screening													✓
		9.2 Fear of results				✓	✓				✓				
		9.3 General worry													
NON COM-B (n=16)		10. Demographics													
		10.1 Sex			✓								✓		
		10.2 Race/ethnicity		✓								✓	✓		
		10.3 Age			✓				✓			✓	✓	✓	
		THEME Subtheme	S27. Raju et al., 2020	S28. Richman et al., 2022	S29. Richmond et al., 2024	S30. Rong et al., 2023	S31. Roth et al., 2018	S32. Sayani et al., 2021	S33. Schiffelbein et al., 2020	S34. Sedani et al., 2021	S35. Sin et al., 2016	S36. Sun et al., 2022	S37. Wong et al., 2024	S38. Zahnd & Eberth, 2019	S39. Zhao et al., 2021
CAPABILITY (n=31)	Physical Capability	1. Health Status													
		1.1 Comorbidity	✓							✓			✓	✓	
		1.2 Functional limitations	✓					✓							
	Psychological Capability	2. Knowledge & Skills													
		2.2 Good understanding			✓	✓									
		2.3 Low health literacy		✓			✓		✓		✓		✓		
		2.4 Misconceptions	✓	✓					✓		✓		✓		
OPPORTUNITY (n=34)	Physical Opportunity	3. Affordability & Cost Coverage													
		3.1 Insurance limitations	✓												
		3.2 Insurance support			✓							✓		✓	✓
		3.3 Low socioeconomic status													
		3.4 Out-of-pocket cost			✓						✓	✓			✓
		3.5 Financial stability						✓					✓		✓
		4. Screening system structure													
		4.1 Pathway barriers	✓	✓											✓
		4.2 Convenient location													✓
		4.3 Distance burden	✓												
		4.4 Easy scheduling & navigation													
		4.5 Transport & logistical barriers			✓				✓						
		4.6 Transport & logistical support													
		5. Decision Support													
		5.1 Effective outreach													
		5.2 Clear information													
	Social Opportunity	5.3 No clinician recommendation									✓				
		5.4 Shared decision-making			✓			✓							
		5.5 Clinician recommendation		✓	✓		✓	✓	✓		✓		✓		
		6. Cultural & Social Context													
		6.1 Community engagement									✓				
		6.2 Cultural beliefs			✓										
		6.3 Family influence					✓				✓		✓		
MOTIVATION (n=35)	Reflective Motivation	7. Beliefs & Perceptions													
		7.1 Risk awareness				✓	✓		✓		✓				✓
		7.2 Perceived benefits		✓	✓		✓	✓	✓						
		7.3 Health history				✓							✓		
		7.4 Stigma & blame													
		7.5 Healthcare mistrust		✓	✓			✓							
		7.6 Low self-perceived risk											✓		
		8. Health & Lifestyle Behaviors													
		8.1 Preventive health habits								✓	✓				✓
		8.2 Competing priorities		✓	✓			✓			✓		✓		
	Automatic Motivation	8.3 Current smoking	✓			✓							✓		
		8.4 Former smoking											✓		
		9. Emotion-Based													
		9.1 Anxiety about screening		✓	✓		✓								
		9.2 Fear of results	✓	✓					✓						
		9.3 General worry				✓									
NON COM-B (n=16)		10. Demographics													
		10.1 Sex	✓												
		10.2 Race/ethnicity													
		10.3 Age								✓				✓	✓
*Notes: COM-B = Capability–Opportunity–Motivation–Behavior framework.*															

Notes: COM-B = Capability–Opportunity–Motivation–Behavior framework.

### 3.2 Capability-related factors

Physical capability factors were less frequently reported and primarily related to underlying health status. Comorbidity (*n* = 7) was associated with higher screening participation in several contexts, often through heightened perceived vulnerability or healthcare contact. In contrast, functional limitations (*n* = 6), including mobility impairments, chronic illness-related fatigue, mental health burden, or cognitive load that complicated appointment scheduling and follow-through, were associated with lower participation in some settings.

Psychological capability factors were most frequently related to knowledge and understanding. Lower health literacy was reported in 11 studies and included limited awareness of screening, confusion about eligibility and pathways, and uncertainty about what LDCT involves. Misconceptions (*n* = 13) included perceiving screening as symptom-driven, conflating LDCT with other tests, or viewing screening as a one-time event. These factors were generally associated with lower participation.

### 3.3 Opportunity-related factors

Affordability and cost coverage were recurring factors. Insurance coverage limitations (*n* = 5) and out-of-pocket costs (*n* = 9) were associated with lower participation, including concerns about direct or downstream costs. These cost-related constraints were particularly visible in North American studies, while stated-preference evidence from other regions also showed cost sensitivity. Insurance support or insurance-supported pathways (*n* = 8) and indicators of financial stability (n = 8) were associated with higher participation, whereas lower socioeconomic status was linked to reduced uptake (*n* = 7), often interacting with other structural constraints.

Screening system and logistical factors were frequently reported. Scheduling and pathway difficulties (*n* = 6), including fragmented pathways and complex processes, were associated with lower participation, as were distance or travel burden (*n* = 5) and transport or logistical difficulties (n = 5). In contrast, convenient location or proximity (n = 5), easy scheduling and navigation (n = 7), and transport or logistical support (*n* = 3) were associated with higher participation, reflecting the importance of integrated and accessible pathways.

Provider engagement was consistently associated with higher participation. Clinician recommendation (*n* = 12) was associated with participation and often acted as a key access point to screening, particularly in healthcare systems where eligibility assessment requires clinical evaluation. Conversely, absence of clinician recommendation (n = 3) was associated with lower participation. Shared decision-making (*n* = 5) supported participation, particularly when communication was empathic and non-judgmental. Communication supports also included clear information and educational materials (*n* = 7) and effective outreach (*n* = 4).

Community engagement and outreach (n = 4) was associated with higher participation, particularly in programs using culturally tailored approaches, such as community-based communication strategies, trusted local intermediaries, or messaging adapted to community norms. Cultural beliefs and norms (n = 4) showed mixed or context-dependent associations, depending on whether they supported preventive engagement or discouraged discussion of cancer or health. Family influence was common (*n* = 9) and also showed mixed direction: encouragement and shared experiences could motivate screening, whereas discouragement or lack of support could inhibit engagement.

### 3.4 Motivation-related factors

Reflective motivation factors were among the most frequently reported. Perceived benefits of early detection (*n* = 13) were associated with higher participation, with screening viewed as protective, life-saving, or enabling earlier treatment. Awareness of personal risk of lung cancer was also common (*n* = 15) and was reinforced by smoking history, other risk exposures such as occupational exposure or second-hand smoke, and previous medical advice or clinical prompts. In some studies, perceived vulnerability was further shaped by personal or family experiences with cancer or smoking-related disease, increasing perceived susceptibility and interest in screening.

Factors associated with lower participation included low self-perceived risk of lung cancer (*n* = 5), often linked to feeling healthy or underestimating smoking-related risk. Mistrust of healthcare systems (*n* = 6) included institutional distrust, perceived discrimination within healthcare settings, and previous negative experiences with care. Internalized stigma and blame appeared less frequently (*n* = 4) but were described as meaningful factors limiting participation in qualitative accounts.

Health and lifestyle factors showed mixed patterns. Current smoking was frequently associated with lower uptake (*n* = 9), reflecting avoidance, stigma, or lower engagement in preventive care in some contexts. However, smoking could also increase perceived risk and willingness to participate depending on context. Former smoking status was more consistently associated with higher participation (*n* = 7), often linked to health orientation or cessation engagement. Preventive health habits were also associated with higher participation (*n* = 7), including self-efficacy and preventive orientation. Competing commitments and priorities (*n* = 12), such as work demands, caregiving responsibilities, financial strain, or competing health conditions, could limit individuals' ability to prioritize screening participation.

Emotions played a major role. Fear of results was commonly reported (*n* = 11), including fear of diagnosis, distress about abnormal findings, and worry about false positives, sometimes leading to avoidance. Anxiety and stress about the screening process were also frequent (n = 7), including anxiety about scanning, follow-up pathways, or cumulative burden. General worry showed mixed patterns (*n* = 4). In some cases, worry contributed to avoidance of screening, whereas in others it appeared to motivate participation by increasing vigilance and the desire for reassurance, particularly in qualitative accounts.

### 3.5 Demographic factors (non-COM-B)

Demographic factors also interacted with screening uptake. Age (*n* = 10) had a varying direction across contexts and outcomes: reduced uptake at very old ages in some organized programs vs increased intention/uptake elsewhere. Sex was reported in 6 studies, also with mixed direction depending on setting and outcome definition. Race/ethnicity was reported in 5 studies, with evidence of lower uptake among certain minority groups, particularly Black/African American and Hispanic populations in United States-based studies, compared to White populations.

### 3.6 Synthesis of results across COM-B domains

Overall, factors most frequently associated with higher participation clustered within Reflective Motivation, particularly risk awareness (*n* = 15) and perceived benefits of screening (*n* = 13). In contrast, factors frequently associated with lower participation included psychological capability factors such as misconceptions about screening (n = 13) and low health literacy (*n* = 11), alongside reflective motivational factors such as competing priorities (*n* = 12). Several factors showed context-dependent or mixed associations across studies, notably age, sex, family influence, and current smoking status. These factors were therefore not classified as consistently associated with either higher or lower participation (Table 3). A summary of the most frequently reported factors across studies is provided in Table 4.

**Table 4:** Most frequently reported factors associated with higher or lower lung cancer screening participation among screening-eligible adults, organized by Capability–Opportunity–Motivation–Behavior framework domain and subdomain.

Rank	Factor	COM-B domain → subdomain	n studies
	Associated with higher participation		
1	Risk awareness	MOTIVATION → Reflective Motivation	15
2	Perceived benefits	MOTIVATION → Reflective Motivation	13
3	Clinician recommendation	OPPORTUNITY → Social Opportunity	12
4	Good understanding	CAPABILITY → Psychological Capability	9
5	Insurance support	OPPORTUNITY → Physical Opportunity	8
	Associated with lower participation		
1	Misconceptions	CAPABILITY → Psychological Capability	13
2	Competing priorities	MOTIVATION → Reflective Motivation	12
3	Low health literacy	CAPABILITY → Psychological Capability	11
4	Fear of results	MOTIVATION → Automatic Motivation	11
5	Out-of-pocket cost	OPPORTUNITY → Physical Opportunity	9

Notes: n = number; COM-B = Capability–Opportunity–Motivation–Behavior framework.

Overall, participation in lung cancer screening was shaped by interacting determinants across Capability, Opportunity, and Motivation domains. Notably, several studies reported positive attitudes toward screening alongside practical, informational, or emotional barriers that limited participation, suggesting a gap between favorable screening intentions and actual uptake.

## 4 Discussion

This scoping review synthesized evidence on participation factors in LDCT lung cancer screening among screening-eligible individuals across diverse contexts. Building on the synthesis of factors across COM-B domains presented in the Results, this review integrates heterogeneous evidence within a behavioral framework, highlighting how participation emerges from interacting Capability, Opportunity, and Motivation determinants rather than from isolated barriers. In doing so, it complements prior reviews by organizing determinants within a behavioral framework designed to support intervention development (Cavers et al., 2022; Symvoulakis et al., 2025). It provides a theory-informed foundation for designing screening programs that address behavioral, structural, and relational barriers simultaneously.

Three interpretations emerge. First, a persistent intention–action gap was evident: favorable attitudes toward early detection did not reliably translate into participation when individuals faced practical barriers, fear, uncertainty, or limited support at key steps of the screening pathway. Second, limited understanding and misconceptions regarding eligibility, benefits, and harms increased uncertainty, anxiety, and decisional burden, amplifying other barriers rather than acting in isolation. Third, Opportunity-related determinants, such as downstream costs, accessibility, scheduling, invitations, and reminders, played a central role and interacted with motivational drivers, either reinforcing disengagement or enabling uptake when supportive systems were in place.

Across studies, positive attitudes were insufficient to ensure participation. Although perceived benefits were common, uptake depended on feasibility, emotional readiness, and trust in the screening pathway. COM-B helps explain this pattern: limited understanding increases cognitive burden (Capability), access constraints impede action (Opportunity), and fear, stigma, or mistrust can override perceived benefit under uncertainty (Motivation) (Michie et al., 2011). This aligns with broader screening evidence showing that intentions often fail to translate into action when pathways are complex or poorly supported (Sheeran and Webb, 2016; Meissner et al., 2012). Educational or decision-support interventions alone show limited effects on uptake, whereas interventions that also reduce logistical barriers (e.g., facilitated scheduling, reminders, structured follow-up) are more likely to increase participation (Symvoulakis et al., 2025; Núñez et al., 2024), supporting the need for multi-component approaches acting across COM-B domains.

Low health literacy and misconceptions were common, particularly regarding eligibility criteria, test purpose, harms, and the need for repeated screening. While these gaps did not necessarily preclude positive attitudes, they increased uncertainty and decisional burden, reducing participation (Michie et al., 2011; Jallow et al., 2022). Capability therefore appears necessary but not sufficient: plain-language information may reduce confusion, but without simplified pathways, reminders, or relational support, gains rarely translate into action (Núñez et al., 2024). Interventions combining tailored information with practical facilitation (e.g., structured invitations, reminders, assistance with next steps) may be more effective, especially for populations with lower literacy (Berkman et al., 2011; Sørensen et al., 2012).

Opportunity-related determinants were frequently reported, highlighting the importance of structural and organizational conditions. Cost, insurance limitations, distance, transportation, and scheduling complexity were common factors associated with lower participation and were often cumulative. Conversely, well-organized services, easy scheduling, reminders, and proactive invitations facilitated participation, underscoring service design as a form of behavior change. Evidence from community-based and primary-care–embedded models supports organizational approaches such as centralized invitations, reminders, facilitated scheduling, and coordinated follow-up (Crosbie et al., 2019). These strategies primarily act on Opportunity while also reducing cognitive and emotional burden, and may help reduce inequalities in access (Freeman and Rodriguez, 2011; Peters et al., 2014).

Clinician recommendation was consistently associated with higher participation, while absence of clinician recommendation was associated with lower participation; however, effectiveness depended on how communication was delivered. Empathic, non-judgmental communication and repeated shared decision-making encounters helped address fear, stigma, and misconceptions. This aligns with shared decision-making literature suggesting that recommendation strength alone is insufficient: communication quality, relational trust, and decision-support design are critical, particularly for individuals with lower health literacy (Jallow et al., 2022; Elwyn et al., 2012; McCaffery et al., 2013). Workflow-integrated approaches appear more promising than one-off conversations, although comparative evidence remains limited (Núñez et al., 2024).

Fear and anxiety were important factors associated with lower participation, extending beyond fear of cancer to concerns about false positives, overdiagnosis, and uncertainty. Anticipated judgement related to smoking and mistrust of healthcare systems further reduced engagement, particularly among socio-economically disadvantaged groups. Although higher perceived risk sometimes facilitated participation, risk awareness alone was insufficient when fear or mistrust remained unaddressed; messaging focused solely on risk or benefits rarely changes behavior (Norman et al., 2020; Núñez et al., 2024). These findings align with sociological models in which anticipated judgement and institutional mistrust shape preventive engagement despite perceived benefit (Link and Phelan, 2001), and with broader evidence linking trust in healthcare systems to preventive uptake, especially among structurally disadvantaged populations (Armstrong et al., 2006).

Together with implementation-oriented evidence (Cavers et al., 2022; Symvoulakis et al., 2025; Jallow et al., 2022), these findings suggest participation is unlikely to be improved through information provision alone. Determinants spanned all COM-B domains, indicating the intention–behavior gap reflects interacting Capability, Opportunity, and Motivation constraints; single-component interventions (e.g., eligibility awareness) may have limited impact when structural, relational, or emotional barriers persist. Capability barriers point to clear, accessible, health-literacy–adapted communication about eligibility, procedures, and follow-up. Opportunity determinants emphasize pathway design (cost coverage, scheduling complexity, distance, administrative burden). Motivation-related influences (fear, stigma, competing priorities, trust) support the need for supportive, non-judgmental, culturally sensitive engagement.

Overall, lung cancer screening programs may benefit from approaches that reduce decisional burden, lower practical barriers, and support engagement across the pathway. This aligns with cancer control frameworks cautioning that screening can exacerbate inequities if system-level and relational determinants are not addressed (European Parliament. Directorate General for Parliamentary Research Services, 2025; World Health Organization, 2022; International Agency for Research on Cancer, 2017). While components such as clinician recommendation, reminders, and streamlined pathways appear promising (Núñez et al., 2024), evidence remains limited on how best to combine and adapt these elements across health-system contexts, particularly to address emotional and psychosocial barriers.

A key strength is integrating heterogeneous evidence within COM-B to map interacting determinants across psychological, social, and structural domains, extending prior reviews that primarily enumerate barriers and facilitators. The evidence map enhances transparency by documenting factor recurrence across studies and settings. Limitations include exclusion of grey literature, variable eligibility definitions and screening contexts, geographically uneven evidence, and heterogeneous outcomes. No formal study quality appraisal was conducted, and frequencies should not be interpreted as effect sizes. Affective and relational determinants may also be under-captured in quantitative studies unless explicitly measured.

For policymakers, these findings support integrated strategies that simplify participation pathways while addressing informational and psychosocial barriers. Future research should examine how combinations of interventions reduce the intention–behavior gap and improve equity and explicitly test this gap using experimental designs or retrospective comparisons of willingness versus observed uptake. Priorities include evaluating multi-component strategies (invitations, shared decision-making, reminders, practical support), distinguishing effects across pathway stages (invitation, decision-making, attendance), and assessing performance across socioeconomic and health literacy groups. Further work is needed to understand how fear, stigma, and trust interact with service design and communication; limited theoretical integration and measurement of these mechanisms in trials restrict understanding of why well-designed interventions often yield only modest uptake gains.

## 5 Conclusion

This scoping review shows that participation in lung cancer screening is shaped by interconnected capability, opportunity, and motivation factors. While awareness of risk and perceived benefits of early detection support engagement, participation is frequently constrained by limited health literacy, misconceptions, emotional barriers, and structural constraints related to access, cost, and service organization.

These findings highlight a persistent intention–action gap: favorable attitudes alone are insufficient without clear information, supportive pathways, and trust-building communication. By mapping factors within the COM-B framework, this review offers a structured synthesis to inform the design of equitable, low-friction screening programs. Future research should prioritize multi-component interventions that address behavioral and structural barriers simultaneously and support sustained participation across diverse populations.

## CRediT authorship contribution statement

**Cynthia Schneider:** Writing – review & editing, Writing – original draft, Visualization, Validation, Resources, Project administration, Methodology, Investigation, Funding acquisition, Formal analysis, Data curation, Conceptualization. **Luca Bosso:** Writing – review & editing, Validation, Methodology, Investigation, Formal analysis, Data curation. **Louis Gros:** Writing – review & editing, Validation, Methodology, Formal analysis, Data curation, Investigation. **Marie-Anne Durand:** Writing – review & editing, Validation, Supervision, Methodology, Conceptualization. **Kevin Selby:** Writing – review & editing, Validation, Supervision, Project administration, Methodology, Formal analysis, Conceptualization, Data curation, Investigation. **Mélinée Schindler:** Writing – review & editing, Validation, Conceptualization. **Anna Nicolet:** Writing – review & editing, Validation, Conceptualization. **Joachim Marti:** Writing – review & editing, Validation, Conceptualization. **Romain Freund:** Writing – review & editing, Validation, Conceptualization. **Andrea Bordoni:** Writing – review & editing, Validation, Conceptualization. **Cédric Bongard:** Writing – review & editing, Validation, Conceptualization. **Christina Akre:** Writing – review & editing, Validation, Conceptualization. **Milo Puhan:** Writing – review & editing, Validation, Conceptualization. **Christophe von Garnier:** Writing – review & editing, Validation, Conceptualization. **Jean-Luc Bulliard:** Writing – review & editing, Validation, Conceptualization. **Chiara Pozzessere:** Writing – review & editing, Validation, Conceptualization. **Dominik Menges:** Writing – review & editing, Validation, Conceptualization. **Kevin Ten Haaf:** Writing – review & editing, Validation, Conceptualization.

## Consent for publication

Not applicable.

## Ethics approval and consent to participate

Not applicable. This study is a scoping review of published literature and did not involve human participants or identifiable personal data.

## Funding

This project is supported by funding from the 10.13039/501100004361Swiss Cancer League (Grant No. KLS-6156-08-2024) and Fond'action contre le cancer (Fond'Action Against Cancer - Swiss Cancer Action Fund). The funders had no role in the study design, data collection, analysis, interpretation, or decision to submit the manuscript.

## Declaration of competing interest

The authors declare that they have no known competing financial interests or personal relationships that could have appeared to influence the work reported in this paper.

## Data Availability

No data was used for the research described in the article.

## Appendix A Supplementary data

Supplementary material: Supplementary File S1, PRISMA Extension for Scoping Reviews (PRISMA-ScR) checklist; Supplementary File S2, complete MEDLINE electronic search strategy used for the scoping review; and Supplementary File S3, separate reference list of the 39 studies included in the scoping review.
