# Supplementary material for: Factors influencing participation in lung cancer screening among screening-eligible adults: a scoping review
Source: Prev Med Rep. 2026 Jul 23;69:103585. doi: 10.1016/j.pmedr.2026.103585 (PMC13417980; doi:10.1016/j.pmedr.2026.103585)
Supplement: Supplementary file 1 — Supplementary material: Supplementary File S1, PRISMA Extension for Scoping Reviews (PRISMA-ScR) checklist; Supplementary File S2, complete MEDLINE electronic search strategy used for the scoping review; and Supplementary File S3, separate reference list of the 39 studies included in the scoping review. [file mmc1.docx]

**Factors influencing participation in lung cancer screening among screening-eligible adults: a scoping review**

**Supplementary material**

**Supplementary File S1.** *Preferred Reporting Items for Systematic Reviews and Meta-Analyses extension for Scoping Reviews checklist for the present scoping review of factors influencing participation in lung cancer screening among screening-eligible adults.*

| **SECTION** | **ITEM** | **PRISMA-ScR CHECKLIST ITEM** | **REPORTED ON PAGE #** |
| --- | --- | --- | --- |
| **TITLE** | | | |
| Title | 1 | Identify the report as a scoping review. | 1 |
| **ABSTRACT** | | | |
| Structured summary | 2 | Provide a structured summary that includes (as applicable): background, objectives, eligibility criteria, sources of evidence, charting methods, results, and conclusions that relate to the review questions and objectives. | 2 |
| **INTRODUCTION** | | | |
| Rationale | 3 | Describe the rationale for the review in the context of what is already known. Explain why the review questions/objectives lend themselves to a scoping review approach. | 3 |
| Objectives | 4 | Provide an explicit statement of the questions and objectives being addressed with reference to their key elements (e.g., population or participants, concepts, and context) or other relevant key elements used to conceptualize the review questions and/or objectives. | 4 |
| **METHODS** | | | |
| Protocol and registration | 5 | Indicate whether a review protocol exists; state if and where it can be accessed (e.g., a Web address); and if available, provide registration information, including the registration number. | 4 |
| Eligibility criteria | 6 | Specify characteristics of the sources of evidence used as eligibility criteria (e.g., years considered, language, and publication status), and provide a rationale. | 4-5 |
| Information sources* | 7 | Describe all information sources in the search (e.g., databases with dates of coverage and contact with authors to identify additional sources), as well as the date the most recent search was executed. | 5 |
| Search | 8 | Present the full electronic search strategy for at least 1 database, including any limits used, such that it could be repeated. | 5 |
| Selection of sources of evidence† | 9 | State the process for selecting sources of evidence (i.e., screening and eligibility) included in the scoping review. | 6 |
| Data charting process‡ | 10 | Describe the methods of charting data from the included sources of evidence (e.g., calibrated forms or forms that have been tested by the team before their use, and whether data charting was done independently or in duplicate) and any processes for obtaining and confirming data from investigators. | 6-7 |
| Data items | 11 | List and define all variables for which data were sought and any assumptions and simplifications made. | 6 |
| Critical appraisal of individual sources of evidence§ | 12 | If done, provide a rationale for conducting a critical appraisal of included sources of evidence; describe the methods used and how this information was used in any data synthesis (if appropriate). | Not applicable |
| Synthesis of results | 13 | Describe the methods of handling and summarizing the data that were charted. | 7 |
| **RESULTS** | | | |
| Selection of sources of evidence | 14 | Give numbers of sources of evidence screened, assessed for eligibility, and included in the review, with reasons for exclusions at each stage, ideally using a flow diagram. | 8 |
| Characteristics of sources of evidence | 15 | For each source of evidence, present characteristics for which data were charted and provide the citations. | 8 |
| Critical appraisal within sources of evidence | 16 | If done, present data on critical appraisal of included sources of evidence (see item 12). | Not applicable |
| Results of individual sources of evidence | 17 | For each included source of evidence, present the relevant data that were charted that relate to the review questions and objectives. | 8-12 |
| Synthesis of results | 18 | Summarize and/or present the charting results as they relate to the review questions and objectives. | 12 |
| **DISCUSSION** | | | |
| Summary of evidence | 19 | Summarize the main results (including an overview of concepts, themes, and types of evidence available), link to the review questions and objectives, and consider the relevance to key groups. | 12-14 |
| Limitations | 20 | Discuss the limitations of the scoping review process. | 15 |
| Conclusions | 21 | Provide a general interpretation of the results with respect to the review questions and objectives, as well as potential implications and/or next steps. | 14-16 |
| **FUNDING** | | | |
| Funding | 22 | Describe sources of funding for the included sources of evidence, as well as sources of funding for the scoping review. Describe the role of the funders of the scoping review. | 29 |

*Note: JBI = Joanna Briggs Institute; PRISMA-ScR = Preferred Reporting Items for Systematic reviews and Meta-Analyses extension for Scoping Reviews./ From:* Tricco AC, Lillie E, Zarin W, O'Brien KK, Colquhoun H, Levac D, et al. PRISMA Extension for Scoping Reviews (PRISMAScR): Checklist and Explanation. Ann Intern Med. 2018;169:467–473. [doi: 10.7326/M18-0850](http://annals.org/aim/fullarticle/2700389/prisma-extension-scoping-reviews-prisma-scr-checklist-explanation).

**Supplementary File S2.** *Complete MEDLINE electronic search strategy used for the scoping review of factors influencing participation in lung cancer screening among screening-eligible adults, published between January 2013 and June 2025.*

1. Lung cancer
2. (“Lung Neoplasms”[MeSH] OR lung cancer[tiab] OR pulmonary neoplasm*[tiab] OR lung carcinoma[tiab])
3. Screening / early detection
4. (“Mass Screening”[MeSH] OR “Early Detection of Cancer”[MeSH] OR lung cancer screening[tiab] OR LDCT screening[tiab] OR low-dose CT screening[tiab] OR screening program*[tiab] OR organi?ed screening[tiab])
5. Participation, determinants, and program features
6. (“Patient Participation”[MeSH] OR “Health Behavior”[MeSH] OR “Patient Preference”[MeSH] OR uptake[tiab] OR adherence[tiab] OR acceptability[tiab] OR barrier*[tiab] OR facilitator*[tiab] OR determinant*[tiab] OR preferenc*[tiab] OR decision making[tiab] OR belie*[tiab] OR motivation[tiab] OR invitation*[tiab] OR access*[tiab] OR healthcare access[tiab] OR service delivery[tiab] OR organizational factor*[tiab] OR structural barrier*[tiab] OR screening experience*[tiab])
7. Limit to English or French language, 2013–current
8. Final combination 1 AND 2 AND 3

**Supplementary File S3.** *Separate reference list of the 39 studies included in the scoping review of factors influencing participation in lung cancer screening among screening-eligible adults, published between January 2013 and June 2025.*

| Study ID | Reference |
| --- | --- |
| S1 | Alaniz-Cantú EI, Goodwin K, Smith L, Acosta E, Chávez-Iñiguez A, Evans MJ, et al. Understanding the perceived benefits, barriers, and cues to action for lung cancer screening among Latinos: A qualitative study. Front Oncol. 2024;14:1365739. doi:10.3389/fonc.2024.1365739 PubMed PMID: 38571494; PubMed Central PMCID: PMC10987732. |
| S2 | Ali N, Lifford KJ, Carter B, McRonald F, Yadegarfar G, Baldwin DR, et al. Barriers to uptake among high-risk individuals declining participation in lung cancer screening: a mixed methods analysis of the UK Lung Cancer Screening (UKLS) trial. BMJ Open. 14 juill 2015;5(7):e008254. doi:10.1136/bmjopen-2015-008254 PubMed PMID: 26173719; PubMed Central PMCID: PMC4513485. |
| S3 | Anderson MD, Pickner WJ, Begnaud A. Determinants of Lung Cancer Screening in a Minnesota Urban Indigenous Community: A Community-Based, Participatory, Action-Oriented Study. Cancer Prev Res (Phila). 3 avr 2023;16(4):239-45. doi:10.1158/1940-6207.CAPR-22-0314 PubMed PMID: 36630997; PubMed Central PMCID: PMC10068432. |
| S4 | Cao W, Tan F, Liu K, Wu Z, Wang F, Yu Y, et al. Uptake of lung cancer screening with low-dose computed tomography in China: A multi-centre population-based study. eClinicalMedicine. 1 oct 2022;52. doi:10.1016/j.eclinm.2022.101594 PubMed PMID: 35923428. |
| S5 | Carter-Bawa L, Slaven JE, Monahan PO, Brandzel S, Gao H, Wernli KJ, et al. Unpacking the relationship between shared decision-making and decisional quality, decision to screen, and screening completion in lung cancer screening. Patient Educ Couns. mai 2024;122:108143. doi:10.1016/j.pec.2024.108143 PubMed PMID: 38237528; PubMed Central PMCID: PMC10922311. |
| S6 | Carter-Harris L, Brandzel S, Wernli KJ, Roth JA, Buist DSM. A qualitative study exploring why individuals opt out of lung cancer screening. Fam Pract. 1 avr 2017;34(2):239-44. doi:10.1093/fampra/cmw146 PubMed PMID: 28122849; PubMed Central PMCID: PMC6279209. |
| S7 | Carter-Harris L, Slaven JE, Monahan PO, Shedd-Steele R, Hanna N, Rawl SM. Understanding lung cancer screening behavior: Racial, gender, and geographic differences among Indiana long-term smokers. Prev Med Rep. juin 2018;10:49-54. doi:10.1016/j.pmedr.2018.01.018 PubMed PMID: 29552458; PubMed Central PMCID: PMC5852404. |
| S8 | Cho MK, Cho YH. Factors influencing the intention for lung cancer screening in high-risk populations for lung cancer. Asia Pac J Oncol Nurs. jan 2024;11(1):100332. doi:10.1016/j.apjon.2023.100332 PubMed PMID: 38192279; PubMed Central PMCID: PMC10772583. |
| S9 | Colhoun SR, Parker K, McCook S, Bartholomew K, Baty B, Maxwell A, et al. Perspectives of potentially eligible Indigenous Māori on a lung cancer screening programme: a qualitative study. N Z Med J. 12 avr 2024;137(1593):45-55. doi:10.26635/6965.6335 PubMed PMID: 38603786. |
| S10 | Copeland A, Levy K, Hardy CM, King JC, Rigney M. Influences on Lung Cancer Screening Initiation and Retention in Rural Alabama. J Prim Care Community Health. 2023;14:21501319231168022. doi:10.1177/21501319231168022 PubMed PMID: 37057356; PubMed Central PMCID: PMC10108423. |
| S11 | Couraud S, Greillier L, Brignoli-Guibaudet L, Lhomel C, Viguier J, Morère JF, et al. Current and Former Smokers: Who Wants To Be Screened? Clin Lung Cancer. nov 2018;19(6):493-501. doi:10.1016/j.cllc.2018.07.001 PubMed PMID: 30107977. |
| S12 | Daskalakis C, Shimada A, Myers RE, Eastburn K, DiCarlo MA, Shusted CS, et al. Decision Preferences in Shared Decision-Making for Lung Cancer Screening among White and African American Individuals. Ann Am Thorac Soc. mai 2023;20(5):756-8. doi:10.1513/AnnalsATS.202211-937RL PubMed PMID: 36827233. |
| S13 | Draucker CB, Rawl SM, Vode E, Carter-Harris L. Understanding the decision to screen for lung cancer or not: A qualitative analysis. Health Expect. dec 2019;22(6):1314-21. doi:10.1111/hex.12975 PubMed PMID: 31560837; PubMed Central PMCID: PMC6882261. |
| S14 | Dunlop KLA, Marshall HM, Stone E, Sharman AR, Dodd RH, Rhee JJ, et al. Motivation is not enough: A qualitative study of lung cancer screening uptake in Australia to inform future implementation. PLOS ONE. 30 sept 2022;17(9):e0275361. doi:10.1371/journal.pone.0275361 |
| S15 | Gudina AT, Kamen C, Cheruvu VK, Cupertino P, Rivera MP. Understanding Factors Associated with Uptake of Lung Cancer Screening among Individuals at High Risk. J Health Care Poor Underserved. 2023;34(2):719-30. doi:10.1353/hpu.2023.0038 PubMed PMID: 37464528; PubMed Central PMCID: PMC11210810. |
| S16 | Guo LW, Meng QC, Zheng LY, Chen Q, Liu Y, Xu HF, et al. Special issue « The advance of solid tumor research in China »: Participants with a family history of cancer have a higher participation rate in low-dose computed tomography for lung cancer screening. Int J Cancer. 1 jan 2023;152(1):7-14. doi:10.1002/ijc.34010 PubMed PMID: 35362560; PubMed Central PMCID: PMC9790604. |
| S17 | Jung J, Razzak E, Avenido AR, Rashidi A, Jia S, Tran NQ, et al. Patient-Reported Barriers and Preferred Interventions to Improve Lung Cancer Screening Uptake. J Am Coll Radiol. mars 2025;22(3):269-79. doi:10.1016/j.jacr.2024.10.010 PubMed PMID: 40044305; PubMed Central PMCID: PMC12694652. |
| S18 | Kellen E, Gabriels S, Van Hal G, Goossens MC. Lung cancer screening: intention to participate and acceptability among Belgian smokers. Eur J Cancer Prev. 1 nov 2021;30(6):457-61. doi:10.1097/CEJ.0000000000000656 PubMed PMID: 33369949. |
| S19 | Lee J, Kim Y, Suh M, Hong S, Choi KS. Examining the effect of underlying individual preferences for present over future on lung cancer screening participation: a cross-sectional analysis of a Korean National Cancer Screening Survey. BMJ Open. 23 juill 2020;10(7):e035495. doi:10.1136/bmjopen-2019-035495 PubMed PMID: 32709642; PubMed Central PMCID: PMC7380730. |
| S20 | Li CC, Matthews AK, Kao YH, Lin WT, Bahhur J, Dowling L. Examination of the Association Between Access to Care and Lung Cancer Screening Among High-Risk Smokers. Front Public Health. 2021;9:684558. doi:10.3389/fpubh.2021.684558 PubMed PMID: 34513780; PubMed Central PMCID: PMC8424050. |
| S21 | Lin YA, Lin X, Li Y, Wang F, Arbing R, Chen W, et al. Screening behaviors of high-risk individuals for lung cancer: A cross-sectional study. Asia Pac J Oncol Nurs. avr 2024;11(4):100402. doi:10.1016/j.apjon.2024.100402 PubMed PMID: 38495639; PubMed Central PMCID: PMC10944110. |
| S22 | Navuluri N, Lanford T, Shapiro A, Krishnan G, Johnson AB, Riley IL, et al. Barriers and Facilitators Impacting Lung Cancer Screening Uptake Among Black Veterans: A Qualitative Study. J Natl Compr Canc Netw. 18 avr 2024;22(4):231-6. doi:10.6004/jnccn.2023.7098 PubMed PMID: 38640946; PubMed Central PMCID: PMC11392566. |
| S23 | Navuluri N, Morrison S, Green CL, Woolson SL, Riley IL, Cox CE, et al. Racial Disparities in Lung Cancer Screening Among Veterans, 2013 to 2021. JAMA Netw Open. 1 juin 2023;6(6):e2318795. doi:10.1001/jamanetworkopen.2023.18795 PubMed PMID: 37326987; PubMed Central PMCID: PMC10276308. |
| S24 | Neslund-Dudas C, Tang A, Alleman E, Zarins KR, Li P, Simoff MJ, et al. Uptake of Lung Cancer Screening CT After a Provider Order for Screening in the PROSPR-Lung Consortium. J Gen Intern Med. feb 2024;39(2):186-94. doi:10.1007/s11606-023-08408-9 PubMed PMID: 37783984; PubMed Central PMCID: PMC10853157. |
| S25 | Niranjan SJ, Rivers D, Ramachandran R, Murrell Je, Curry KC, Mubasher M, et al. Disparities in lung cancer screening utilization at two health systems in the Southeastern USA. Cancer Causes and Control. feb 2025;36(2):135-45. doi:10.1007/s10552-024-01929-6 |
| S26 | Quaife SL, Waller J, Dickson JL, Brain KE, Kurtidu C, McCabe J, et al. Psychological Targets for Lung Cancer Screening Uptake: A Prospective Longitudinal Cohort Study. J Thorac Oncol. dec 2021;16(12):2016-28. doi:10.1016/j.jtho.2021.07.025 PubMed PMID: 34403828. |
| S27 | Raju S, Khawaja A, Han X, Wang X, Mazzone PJ. Lung Cancer Screening: Characteristics of Nonparticipants and Potential Screening Barriers. Clin Lung Cancer. sept 2020;21(5):e329-36. doi:10.1016/j.cllc.2019.11.016 PubMed PMID: 32371161. |
| S28 | Richman IB, Prasad TV, Gross CP. Lost to follow up?: A qualitative study of why some patients do not pursue lung cancer screening. Prev Med Rep. oct 2022;29:101909. doi:10.1016/j.pmedr.2022.101909 PubMed PMID: 35911579; PubMed Central PMCID: PMC9326340. |
| S29 | Richmond J, Fernandez JR, Bonnet K, Sellers A, Schlundt DG, Forde AT, et al. Patient Lung Cancer Screening Decisions and Environmental and Psychosocial Factors. JAMA Netw Open. 1 mai 2024;7(5):e2412880. doi:10.1001/jamanetworkopen.2024.12880 PubMed PMID: 38819825; PubMed Central PMCID: PMC11143466. |
| S30 | Rong F, Shi R, Hu L, Wang D, Lv X, Zhao Y. Impact of risk perception and disease cognition on the willingness to participate in screening for lung cancer in a high-risk population. Eur J Cancer Prev. 1 mars 2024;33(2):141-51. doi:10.1097/CEJ.0000000000000843 PubMed PMID: 37751366. |
| S31 | Roth JA, Carter-Harris L, Brandzel S, Buist DSM, Wernli KJ. A qualitative study exploring patient motivations for screening for lung cancer. PLOS ONE. juil 2018;13(7):e0196758. doi:10.1371/journal.pone.0196758 |
| S32 | Sayani A, Vahabi M, O’Brien MA, Liu G, Hwang S, Selby P, et al. Advancing health equity in cancer care: The lived experiences of poverty and access to lung cancer screening. PLOS ONE. mai 2021;16(5):e0251264. doi:10.1371/journal.pone.0251264 |
| S33 | Schiffelbein JE, Carluzzo KL, Hasson RM, Alford-Teaster JA, Imset I, Onega T. Barriers, Facilitators, and Suggested Interventions for Lung Cancer Screening Among a Rural Screening-Eligible Population. J Prim Care Community Health. 2020;11:2150132720930544. doi:10.1177/2150132720930544 PubMed PMID: 32506999; PubMed Central PMCID: PMC7278309. |
| S34 | Sedani AE, Ford LA, James SA, Beebe LA. Factors associated with low-dose CT lung cancer screening participation in a high burden state: Results from the 2017-2018 BRFSS. J Cancer Policy. juin 2021;28:100284. doi:10.1016/j.jcpo.2021.100284 PubMed PMID: 35559913. |
| S35 | Sin MK, Ha A, Taylor V. Sociocultural Barriers to Lung Cancer Screening Among Korean Immigrant Men. Journal of Community Health. 2016;41(4):790-7. |
| S36 | Sun J, Perraillon MC, Myerson R. The Impact of Medicare Health Insurance Coverage on Lung Cancer Screening. Med Care. 1 jan 2022;60(1):29-36. doi:10.1097/MLR.0000000000001655 PubMed PMID: 34739415; PubMed Central PMCID: PMC8663516. |
| S37 | Wong LY, Choudhary S, Kapula N, Lin M, Elliott IA, Guenthart BA, et al. Barriers to Completing Low Dose Computed Tomography Scan for Lung Cancer Screening. Clin Lung Cancer. juill 2024;25(5):424-30. doi:10.1016/j.cllc.2024.04.014 PubMed PMID: 38749902. |
| S38 | Zahnd WE, Eberth JM. Lung Cancer Screening Utilization: A Behavioral Risk Factor Surveillance System Analysis. Am J Prev Med. août 2019;57(2):250-5. doi:10.1016/j.amepre.2019.03.015 PubMed PMID: 31248742. |
| S39 | Zhao Z, Du L, Wang L, Wang Y, Yang Y, Dong H. Preferred Lung Cancer Screening Modalities in China: A Discrete Choice Experiment. Cancers (Basel). 3 dec 2021;13(23):6110. doi:10.3390/cancers13236110 PubMed PMID: 34885217; PubMed Central PMCID: PMC8656503. |
|  |  |
